# Supplementary material for: RBM39 degrader invigorates innate immunity to eradicate neuroblastoma despite cancer cell plasticity
Source: Nat Commun. 2025 Sep 17;16:8287. doi: 10.1038/s41467-025-63979-x (PMC12443969; doi:10.1038/s41467-025-63979-x)

Supplementary Information

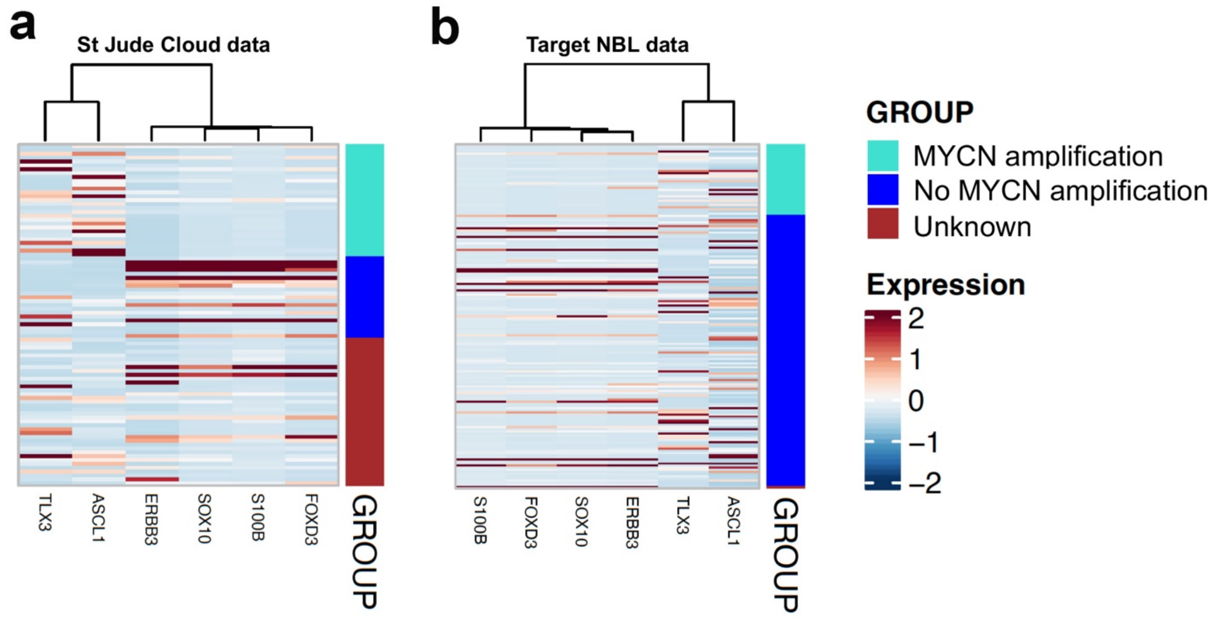

Supplementary Figure 1. Expression of Schwann cell precursor in human neuroblastomas.

Raw RNA-seq data requested from Pediatric Cancer Genome Project (PCGP)[63] (a) and NCI's Therapeutically Applicable Research To Generate Effective Treatments (TARGET, https://ocg.cancer.gov/programs/target)[21] (b) were processed by internal AutoMapper pipeline (described in method section of RNA-seq and analysis). TPM (transcript per million) matrix of neuroblastoma samples from both studies were extract respectively to generate heatmaps for SCP signature. MYCN amplification status were annotated in color bars. Heatmap pseudocolor indicated z-score of log2TPM.

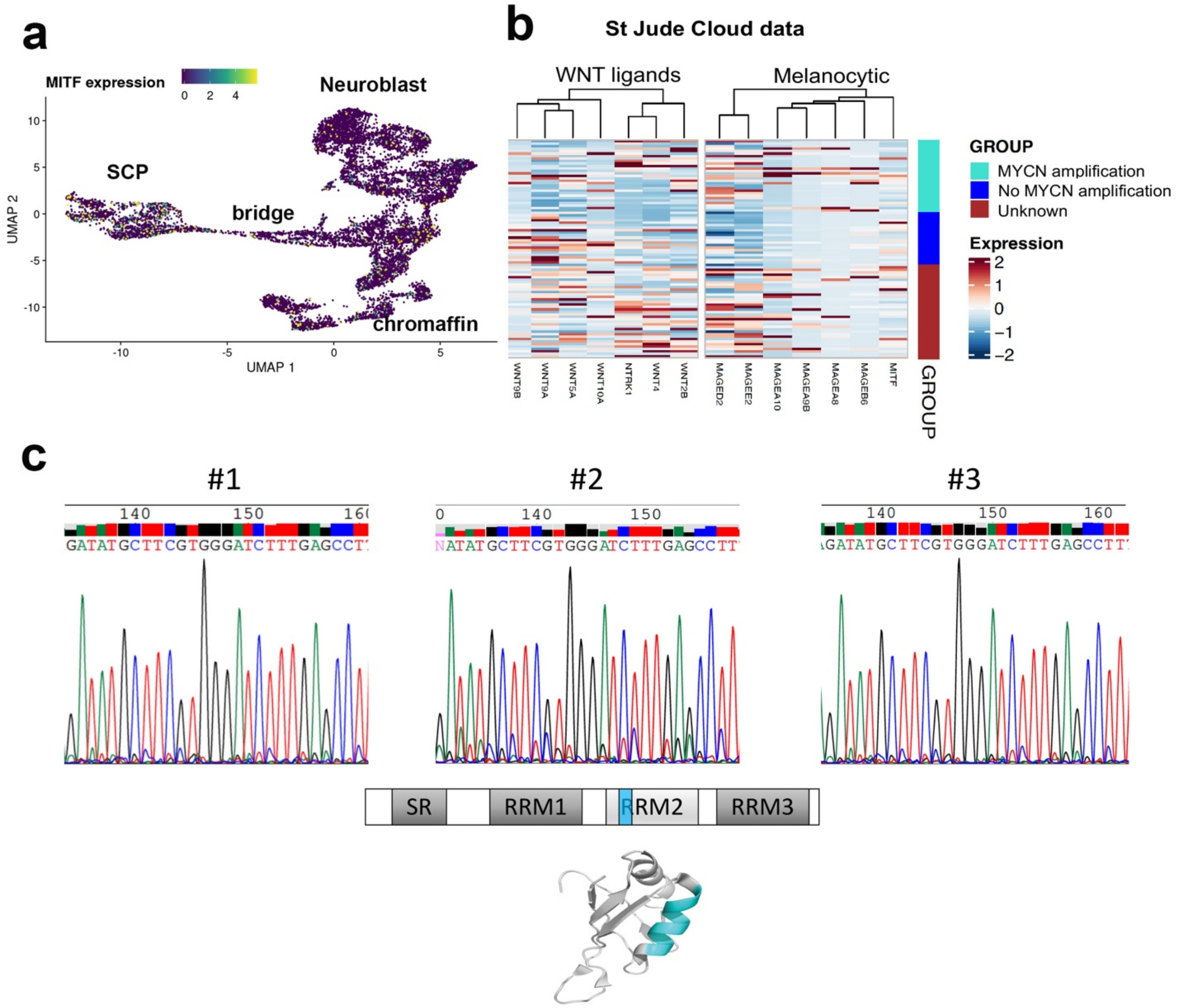

Supplementary Figure 2. Deconvolution of gene signatures in indisulam-resistant SJNB14 PDX. (a) Low *MITF* expression in the SCP cell population, bridge cells, neuroblasts and Chromaffin cells from study[9] (https://adrenal.kitz-heidelberg.de/developmental_programs_NB_viz/). (b) Raw RNA-seq data requested from Pediatric Cancer Genome Project (PCGP) were processed by internal AutoMapper pipeline (described in method section of RNA-seq and analysis). TPM matrix of neuroblastoma samples were extract respectively to generate heatmaps for WNT ligands and melanocytic signature. MYCN amplification status were annotated in color bars. Heatmap pseudocolor indicated z-score of log2TPM. (c) Sanger DNA sequencing verifies that there are no mutations at the hotspot sequence of RBM39 in three resistant tumors. The hotspot was indicated by the blue color in RRM2 motif of RBM39.

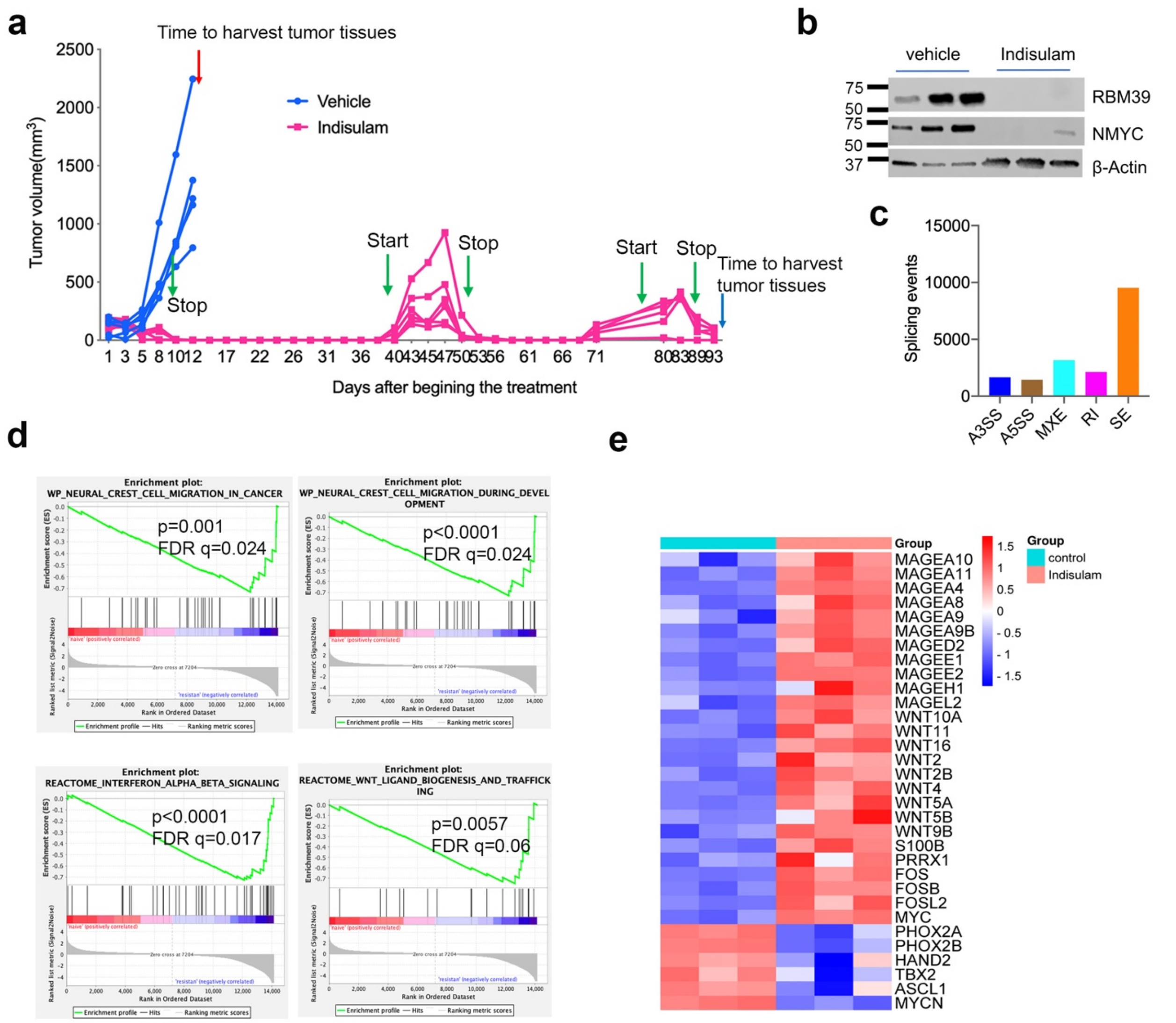

Supplementary Figure 3. SIMA tumor cells undergo cell state alterations during indisulam therapy. (a) Tumor growth curve for SIMA implanted in NSG mice undergoing repeated cycles (2-week as one cycle) of treatment with 25mg/kg indisulam, 5 days on, two ways off. (b) Western blot analysis of SIMA xenograft tissues treated with indisulam during the third cycle of treatment, with indicated antibodies. The experiment was repeated twice with similar results. (c) Alternative splicing events induced by indisulam during third cycle of treatment. (d) GSEA shows the gene signatures significantly upregulated in resistant tumors vs naïve tumors. (e) Heatmap showing that the expression changes of melanocytic markers, Wnt ligands, MES and ADRN transcriptional factors in naïve vs resistant tumors (biological replicates, n=3).

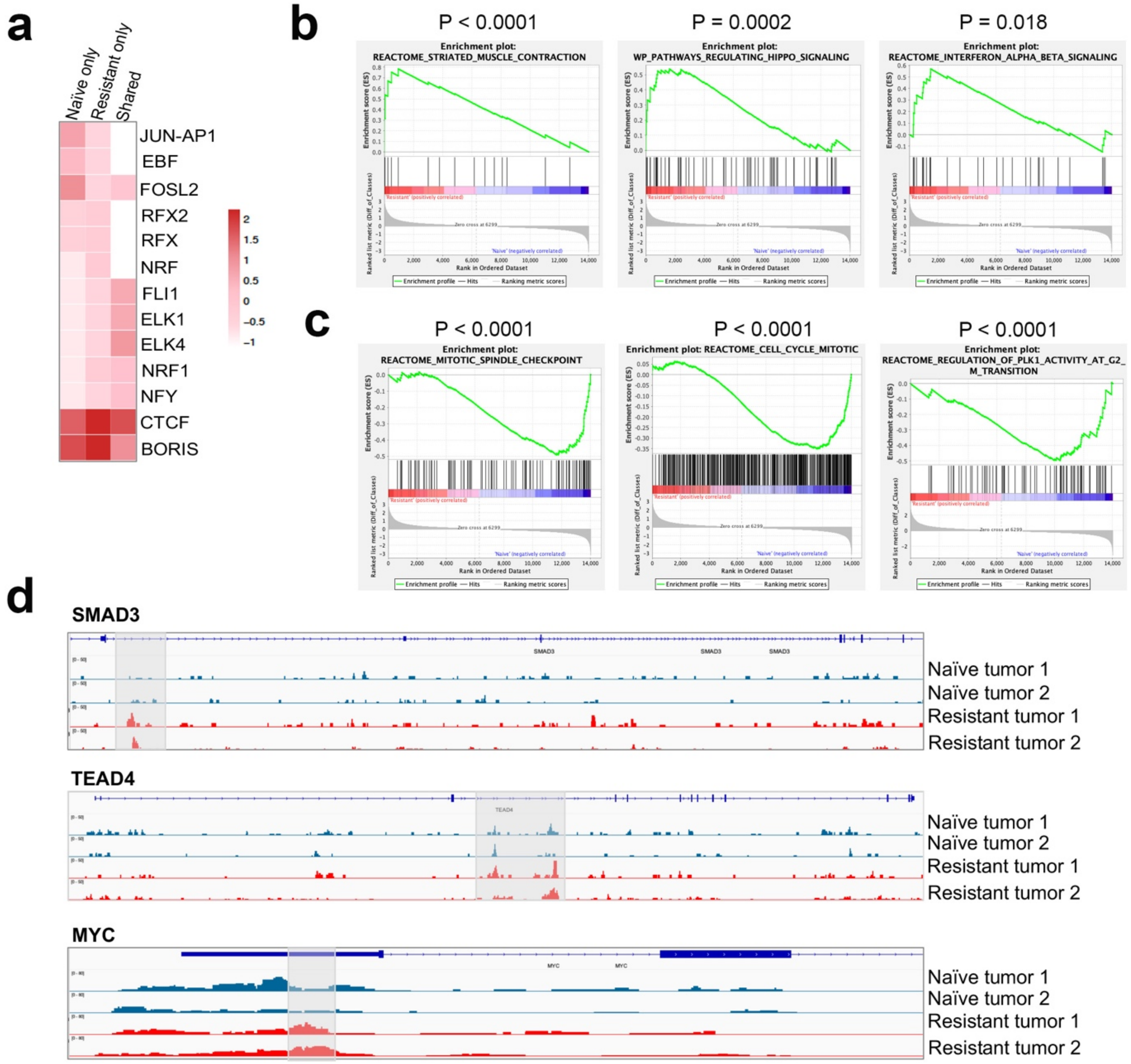

Supplementary Figure 4. Epigenetic reprogramming of MYCN–amplified SJNB14 tumor cells after they developed full resistance to indisulam therapy after repeated treatments. (a) Heatmap indicates the binding motifs for transcriptional factors enriched in naïve, resistant and both based on Homer Motif analysis of H3K27Ac peaks. (b, c) GSEA results for the H3K27Ac upregulated (b) and downregulated (c) at the genes in resistant tumors. (d) Snapshots by IGV showing the H3K27Ac peaks (highlighted in grey color) upregulated at the genomic loci of MES CRC TFs in resistant tumors.

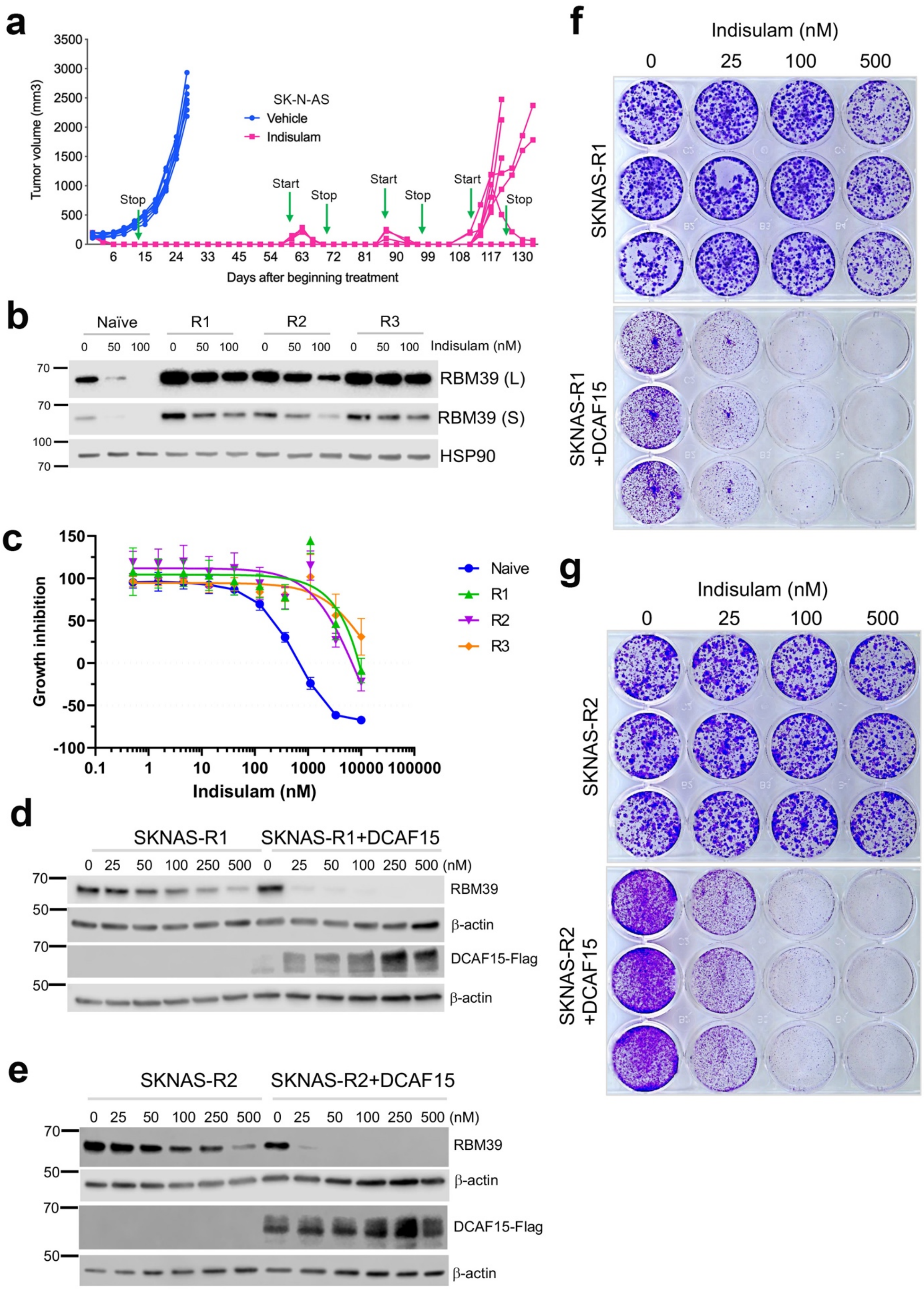
a
SK-N-AS
Vehicle
Indisulam
Tumor volume (mm3)
Days after beginning treatment
Stop
Start
Stop
Start
Stop
Start
Stop
b
Naïve
R1
R2
R3
Indisulam (nM)
RBM39 (L)
RBM39 (S)
HSP90
c
Growth inhibition
Indisulam (nM)
Naive
R1
R2
R3
d
SKNAS-R1
SKNAS-R1+DCAF15
(nM)
RBM39
β-actin
DCAF15-Flag
β-actin
e
SKNAS-R2
SKNAS-R2+DCAF15
(nM)
RBM39
β-actin
DCAF15-Flag
β-actin
f
Indisulam (nM)
0
25
100
500
SKNAS-R1
SKNAS-R1 +DCAF15
g
Indisulam (nM)
0
25
100
500
SKNAS-R2
SKNAS-R2 +DCAF15

Supplementary Figure 5. SK-N-AS tumor cells develop full resistance to indisulam therapy after repeated treatments. (a) Tumor growth curve for SK-N-AS implanted in NSG mice undergoing repeated cycles (2-week as one cycle) of treatment with 25mg/kg indisulam, 5 days on, two ways off. (b) Western blot analysis of SK-N-AS naïve and indisulam-resistant cells treated with different concentrations of indisulam with indicated antibodies. L, long exposure of the gel; s, short exposure of the gel. The experiment was repeated twice with similar results. (c) Growth inhibition curve of SK-N-AS naïve and indisulam-resistant cells assessed by Prestoblue assay (biological replicates n=8 for each time point). Data are presented as Mean +/- SEM. (d, e) Western blot with indicated antibodies to assess the expression of RBM39 in indisulam-resistant SK-N-AS cells (R1 and R2) and the DACF15 overexpressing indisulam-resistant SK-N-AS cells which are treated with different concentrations of indisulam for 24hours. The experiment was repeated twice with similar results. (f, g) Crystal violet staining of indisulam-resistant SK-N-AS cells (R1 and R2) and the DACF15 overexpressing indisulam-resistant SK-N-AS cells which are treated with different concentrations of indisulam for 5 days.

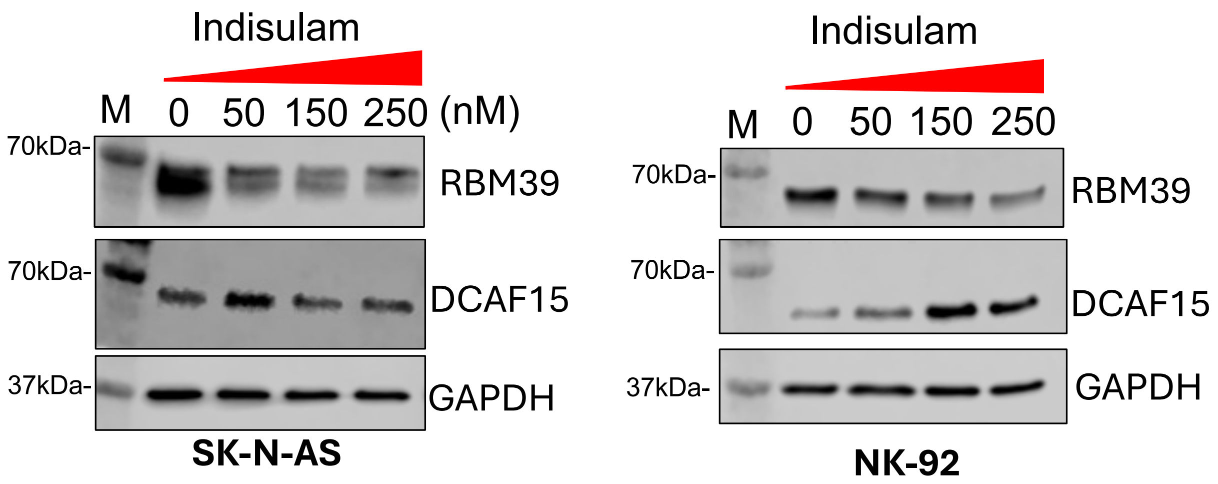

Supplementary Figure 6. Western blot assessment of RBM39 degradation in SK-N-AS and NK92 cells by indisulam.

SK-N-AS and NK92 cells were plated at 2.5×10^5 cells/ well in 1mL volume and allowed to adhere/suspend in wells overnight. The following day cells were treated with indisulam with indicated concentrations for 24hrs. The whole cell lysates were harvested for western blot analysis with indicated antibodies. The experiment was repeated twice with similar results.

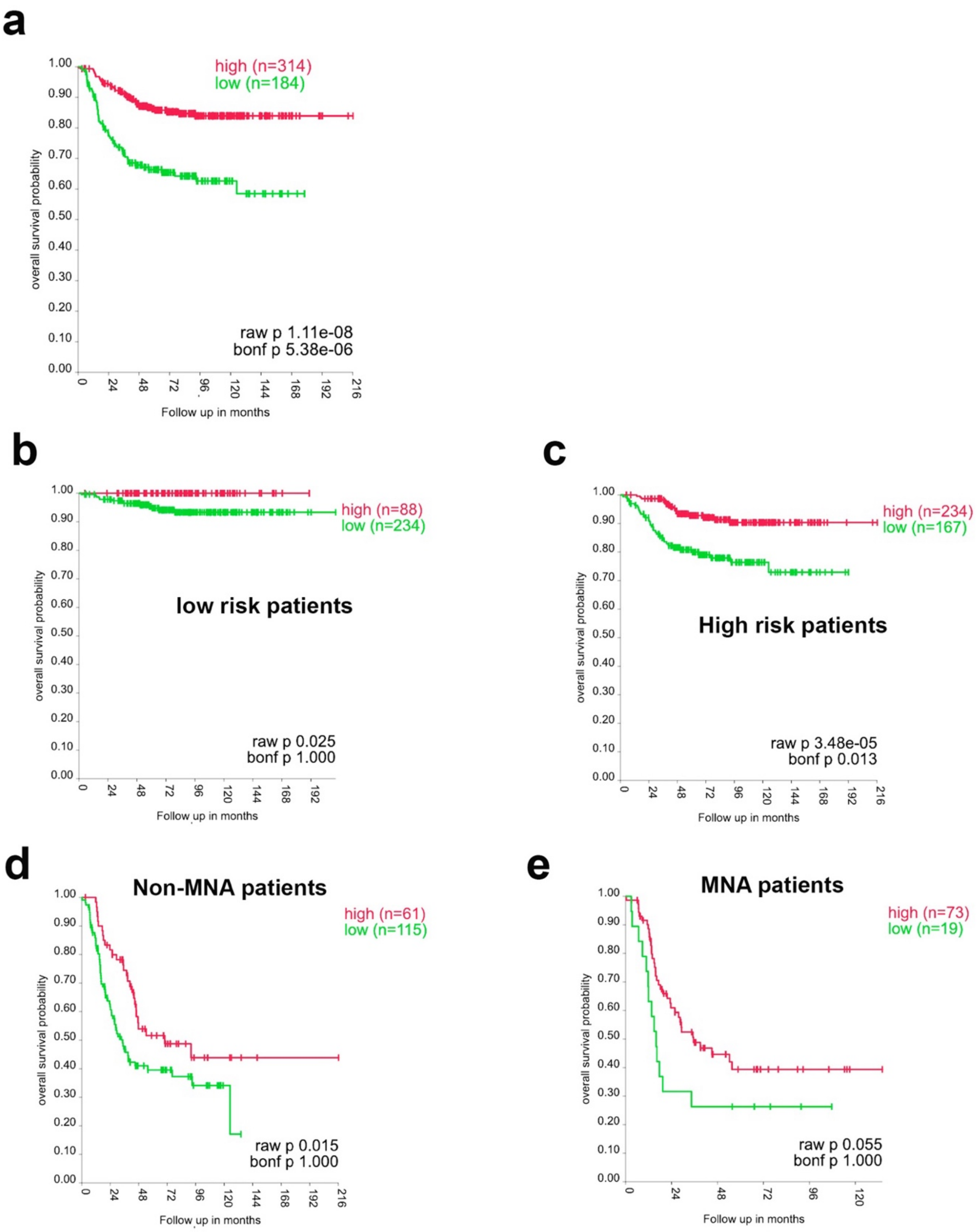

Supplementary Figure 7. High expression of *NCR1* is correlated with a better survival of neuroblastoma patients. (a) Kaplan-Meier overall survival for neuroblastoma patients with high and low expression of *NCR1* gene in SEQC cohort (GSE62564)[83]. (b,c) Kaplan-Meier overall survival for low risk (b) and high risk (c) neuroblastoma patients with high and low expression of *NCR1* gene in SEQC cohort (GSE62564)[83]. (d, e) Kaplan-Meier overall survival for non-MYCN amplified (d) and MYCN amplified (e) neuroblastoma patients with high and low expression of *NCR1* gene in SEQC cohort (GSE62564)[83].

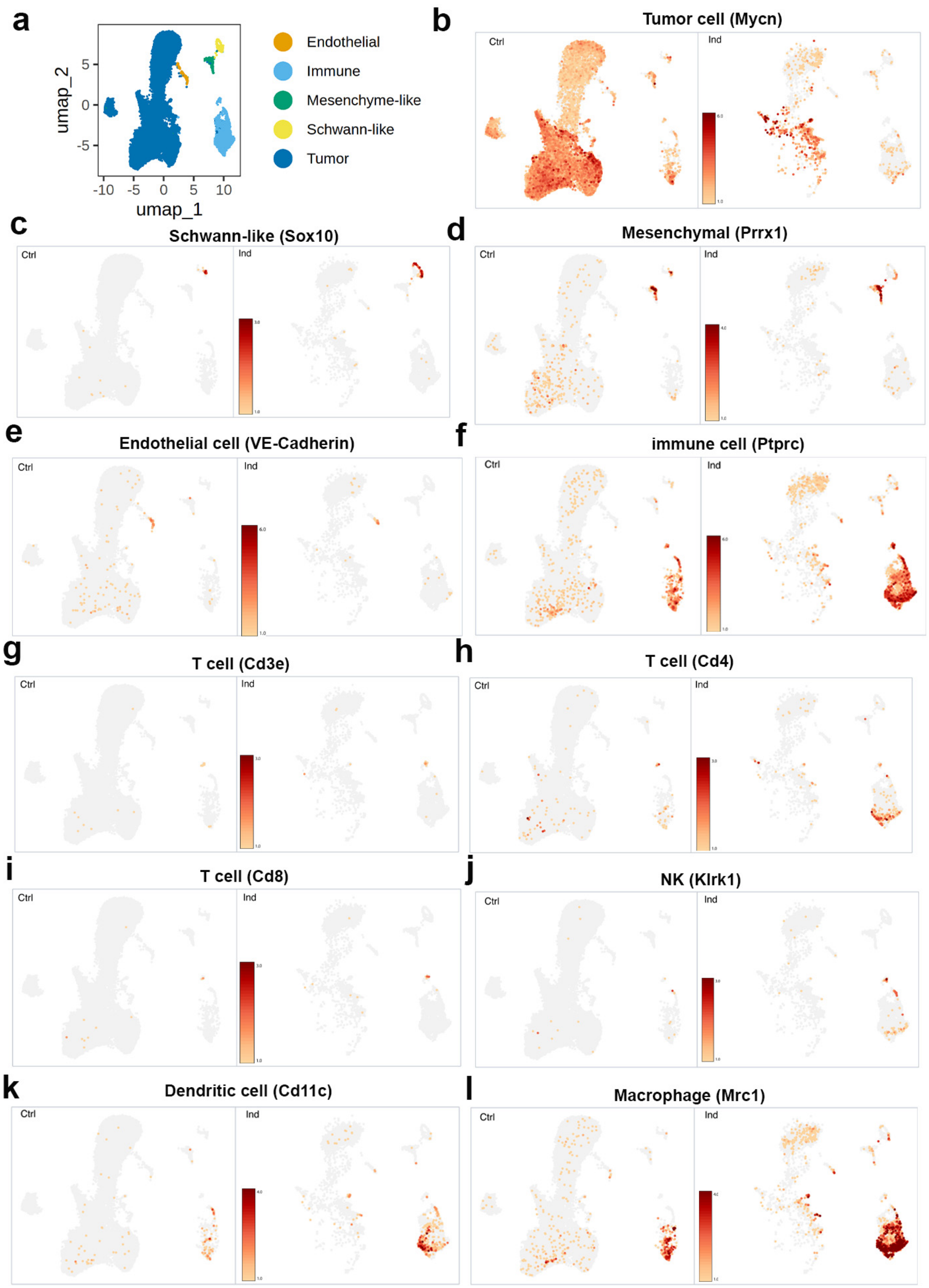

Supplementary Figure 8. Indisuam induces changes in cell composition. (a) UMAP showing 5 major cell types in Th-MYCN/ALK^{F1178L} tumors treated with vehicle control and indisulam (25mg/kg) for 5 days. (b-i) UMAP showing markers for each cell population.

a

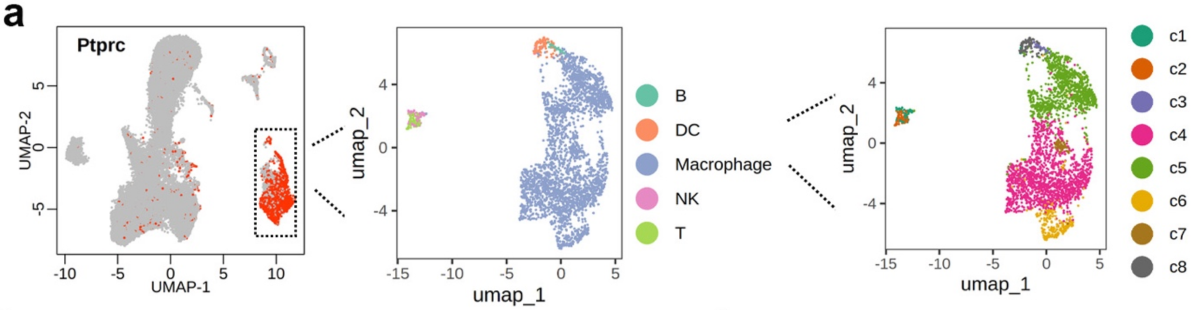

b

cluster	cell type	Ctrl (cell count)	Ind (cell count)	Ctrl(%)	Ind(%)
c1	NK	24	39	0.09%	0.61%
c2	T	14	61	0.05%	0.96%
c3	B	10	19	0.04%	0.03%
c4	DC	24	85	0.09%	1.30%
c5	Macrophage	85	1330	0.33%	20.92%
c6	Macrophage	83	861	0.33%	13.54%
c7	Macrophage	170	97	0.67%	1.53%
c8	Macrophage	78	0	0.31%	0.00%

c

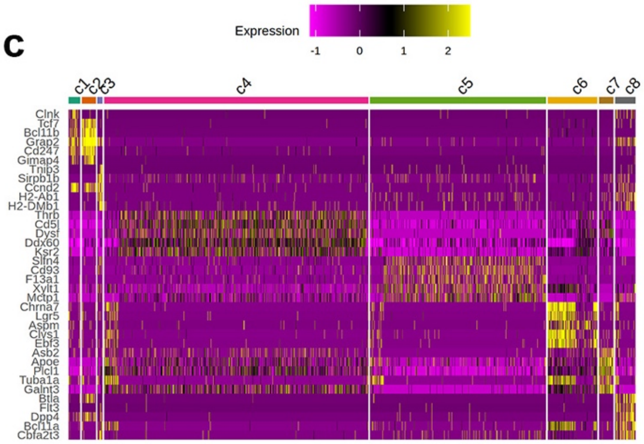

d

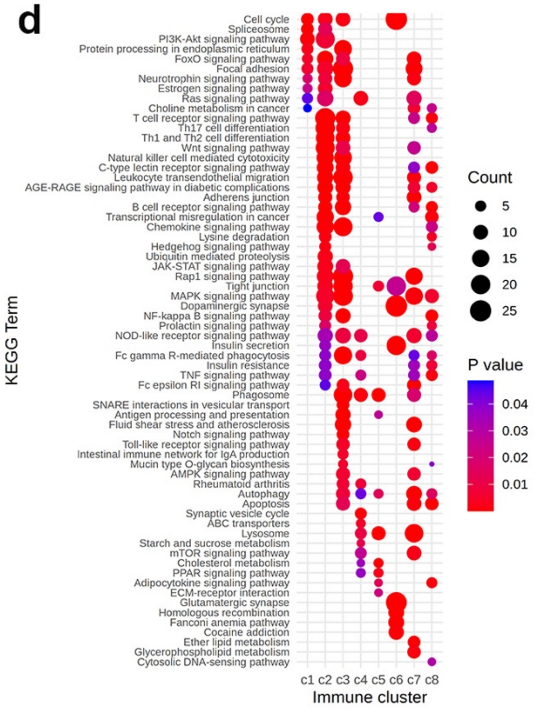

Supplementary Figure 9. Indisuam induces immune cell infiltration. (a) UMAP showing Ptprc (CD45) positive immune cell populations that are futher classified into 8 clusters based on differential gene expression. (b) Summary of cell counts and percentage of each cluster of immune cell type in Th-MYCN/ALK^{F1178L} tumors treated with vehicle control and indisulam (25mg/kg) for 5 days. (c) Heatmap showing the expression of differential genes in each cluster of immune cells. (d) Bubble plot showing the gene set enrichment for each class of immune cells.

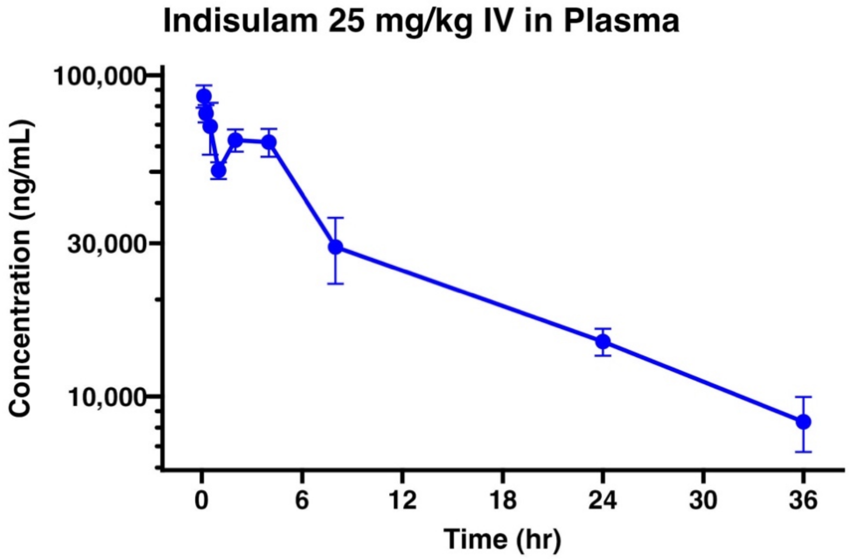

Supplementary Figure 10. Plasma concentration-time profile of indisulam from pharmacokinetic study. Indisulam concentrations in plasma of CB17/SCIG mice were assessed by mass spectrometry over time after a 25 mg/kg bolus tail vein injection of indisulam. Data points are shown as mean and standard deviation. Biological replicates n=3 for each time point. Data are presented as Mean +/- SEM

Uncropped blots for Supplementary Figure 3b

Image ID: 0001102_03
Acquire Time: Oct 26, 2020, 9:12:52 AM

Page 1

Images Table

#	Image ID	Acquire Time	Channels	Integration Times	Analysis	Image Name	Comment
1	0001102_03	Oct 26, 2020, 9:12:52 AM	700, Chemi	04:30 00:30	Manual	0001102_03	RBM39 BE2c RBM39 KD tumor

Image Display Values

Channel	Color	Minimum	Maximum	K
Chemi	Gray Scale (Black on White)	0.000000238	0.000150	0
700	Gray Scale (Black on White)	0.000119	0.0312	0

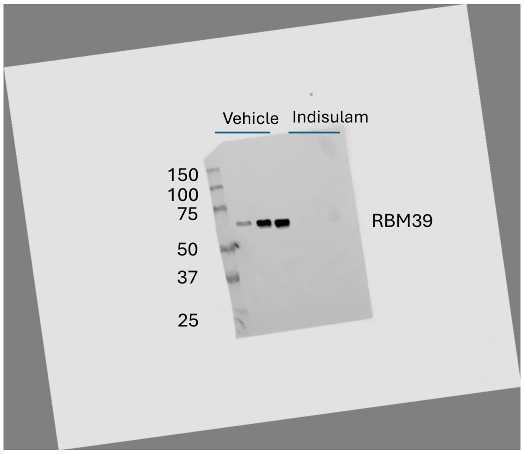

Image ID: 0001101_04
Acquire Time: Oct 26, 2020, 9:06:15 AM

Page 1

Images Table

#	Image ID	Acquire Time	Channels	Integration Times	Analysis	Image Name	Comment
1	0001101_04	Oct 26, 2020, 9:06:15 AM	700, Chemi	04:30 00:30	Manual	0001101_04	NMYC BE2c RBM39 KD tumor

Image Display Values

Channel	Color	Minimum	Maximum	K
Chemi	Gray Scale (Black on White)	0.000000417	0.000275	0
700	Gray Scale (Black on White)	0.000137	0.0176	0

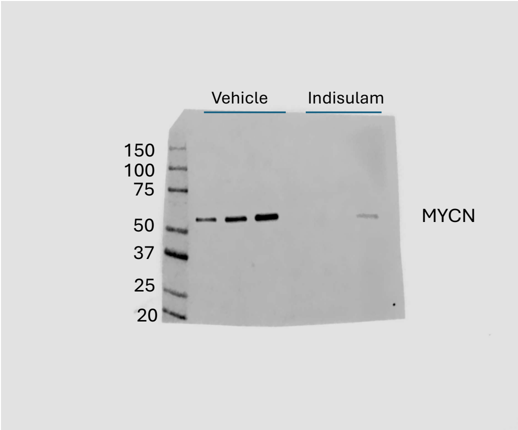

Image ID: 0001099_01
Acquire Time: Oct 26, 2020, 10:28:51 AM

Page 1

Images Table

#	Image ID	Acquire Time	Channels	Integration Times	Analysis	Image Name	Comment	Image Modifications
1	0001099_01	Oct 26, 2020, 10:28:51 AM	700, Chemi		Manual	0001099_01	actin	

Image Display Values

Channel	Color	Minimum	Maximum	K
700	Gray Scale (Black on White)	0.0000359	0.0262	0
Chemi	Gray Scale (Black on White)	0.00000328	0.00112	0

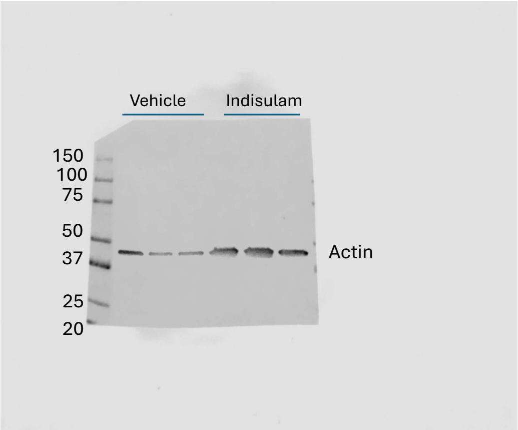

Uncropped blots for Supplementary Figure 5b

Image ID: 0001897_02
Acquire Time: May 26, 2023, 12:59:43 PM

Page 1

Images Table

#	Image ID	Acquire Time	Channels	Integration Times	Analysis	Image Name
1	0001897_02	May 26, 2023, 12:59:43 PM	700, Chemi	02:00 00:30	Manual	0001897_02

Image Display Values

Channel	Color	Minimum	Maximum	K
Chemi	Gray Scale (Black on White)	0.0000141	0.000178	0
700	Gray Scale (Black on White)	0.000120	0.0841	0

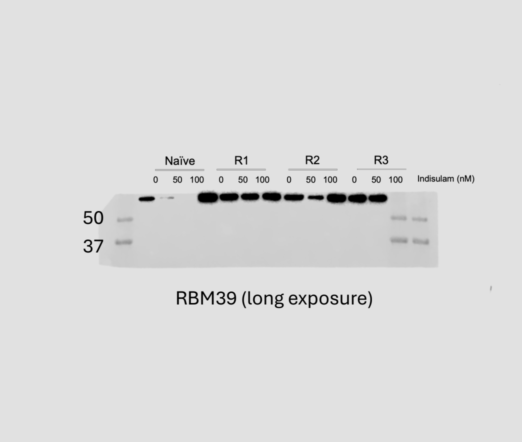

RBM39 (long exposure)

Image ID: 0001897_01
Acquire Time: May 26, 2023, 12:59:43 PM

Page 1

Images Table

#	Image ID	Acquire Time	Channels	Integration Times	Analysis	Image Name
1	0001897_01	May 26, 2023, 12:59:43 PM	700, Chemi	02:00 00:30	Manual	0001897_01

Image Display Values

Channel	Color	Minimum	Maximum	K
Chemi	Gray Scale (Black on White)	0.0000141	0.00170	0
700	Gray Scale (Black on White)	0.000120	0.0841	0

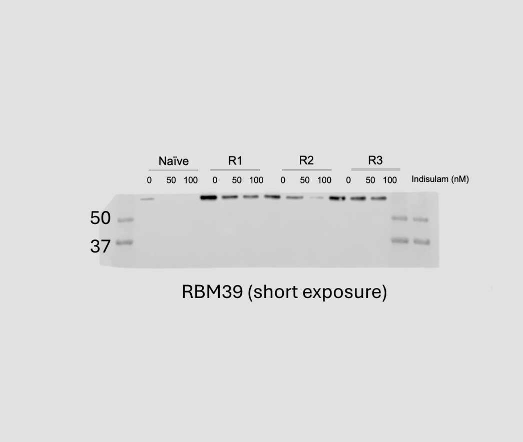

RBM39 (short exposure)

Image ID: 0001898_01
Acquire Time: May 26, 2023, 1:06:01 PM

Page 1

Images Table

#	Image ID	Acquire Time	Channels	Integration Times	Analysis	Image Name
1	0001898_01	May 26, 2023, 1:06:01 PM	700, Chemi	02:00 00:30	Manual	0001898_01

Image Display Values

Channel	Color	Minimum	Maximum	K
Chemi	Gray Scale (Black on White)	0.0217	0.111	0
700	Gray Scale (Black on White)	0.000119	0.173	0

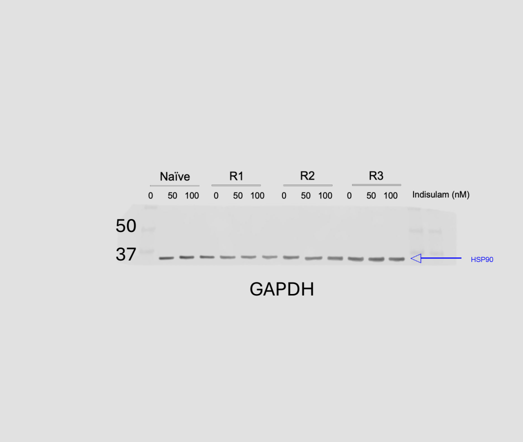

GAPDH

Uncropped blots for Supplementary Figure 5d

Image ID: 0001385_03
Acquire Time: Mar 22, 2021, 8:35:56 AM

Images Table

#	Image ID	Acquire Time	Channels	Integration Times	Analysis	Image Name	Comment
1	0001385_03	Mar 22, 2021, 8:35:56 AM	700, Chemi	01:58 00:30	Manual	0001385_03	RBM39 SKNAS-R1

Image Display Values

Channel	Color	Minimum	Maximum	K
Chemi	Gray Scale (Black on White)	0.00000143	0.00283	0
700	Gray Scale (Black on White)	0.0000445	0.225	0

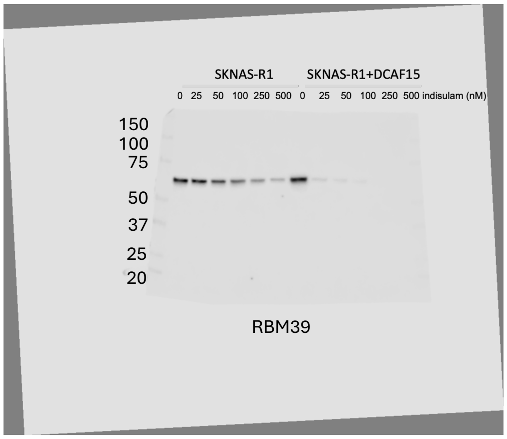

Image ID: 0001388_03
Acquire Time: Mar 23, 2021, 4:46:39 PM

Images Table

#	Image ID	Acquire Time	Channels	Integration Times	Analysis	Image Name	Comment
1	0001388_03	Mar 23, 2021, 4:46:39 PM	700, Chemi	01:58 00:30	Manual	0001388_03	b-actin R1

Image Display Values

Channel	Color	Minimum	Maximum	K
Chemi	Gray Scale (Black on White)	0.00000423	0.00592	0
700	Gray Scale (Black on White)	0.000119	0.0497	0

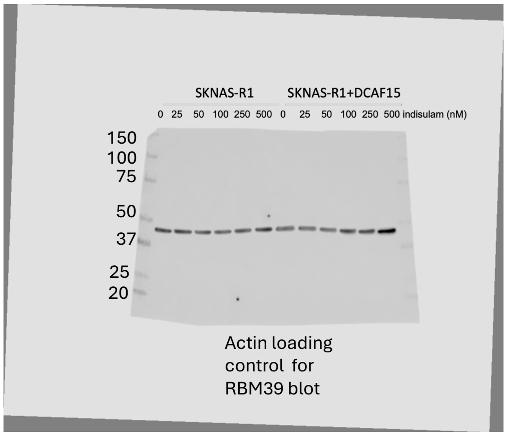

Image ID: 0001380_03
Acquire Time: Mar 19, 2021, 1:17:31 PM

Images Table

#	Image ID	Acquire Time	Channels	Integration Times	Analysis	Image Name	Comment
1	0001380_03	Mar 19, 2021, 1:17:31 PM	700, Chemi	01:58 00:30	Manual	0001380_03	R1 R1+DCAF15 WB for DCAF15

Image Display Values

Channel	Color	Minimum	Maximum	K
Chemi	Gray Scale (Black on White)	0.000000775	0.000581	0
700	Gray Scale (Black on White)	0.0000334	0.0279	0

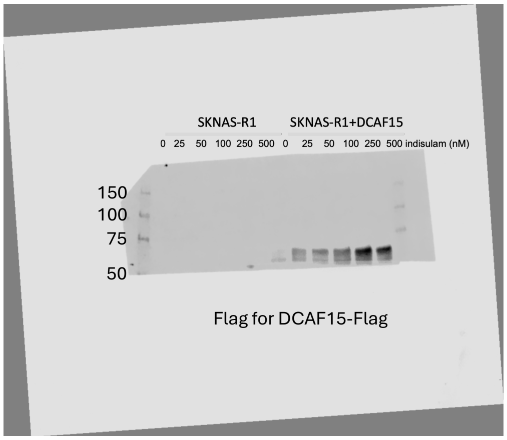

Image ID: 0001378_03
Acquire Time: Mar 19, 2021, 1:03:00 PM

Images Table

#	Image ID	Acquire Time	Channels	Integration Times	Analysis	Image Name	Comment
1	0001378_03	Mar 19, 2021, 1:03:00 PM	700, Chemi	01:58 00:30	Manual	0001378_03	R1 R1+DCAF15 actin

Image Display Values

Channel	Color	Minimum	Maximum	K
Chemi	Gray Scale (Black on White)	0.00000143	0.00457	0
700	Gray Scale (Black on White)	0.0000473	0.193	0

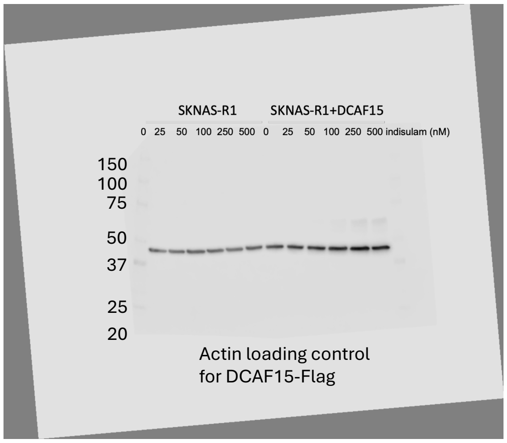

Uncropped blots for Supplementary Figure 5e

Image ID: 0001387_03
Acquire Time: Mar 22, 2021, 8:44:18 AM

Page 1

Images Table

#	Image ID	Acquire Time	Channels	Integration Times	Analysis	Image Name	Comment
1	0001387_03	Mar 22, 2021, 8:44:18 AM	700, Chemi	01:58 00:30	Manual	0001387_03	RBM39 SKNAS-R2

Image Display Values

Channel	Color	Minimum	Maximum	K
Chemi	Gray Scale (Black on White)	0.0000120	0.000738	0
700	Gray Scale (Black on White)	0.0000000596	0.0353	0

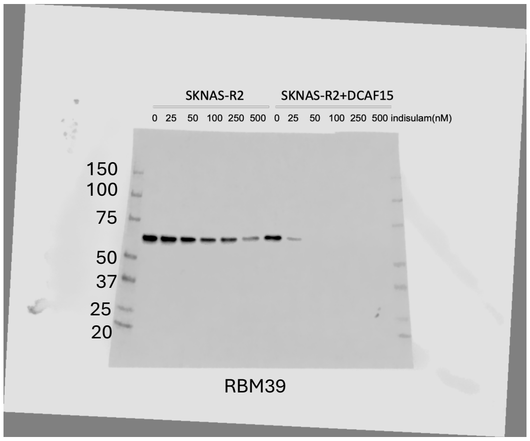

Image ID: 0001389_02
Acquire Time: Mar 23, 2021, 4:51:37 PM

Page 1

Images Table

#	Image ID	Acquire Time	Channels	Integration Times	Analysis	Image Name	Comment
1	0001389_02	Mar 23, 2021, 4:51:37 PM	700, Chemi	01:58 00:30	Manual	0001389_02	b-actin R2

Image Display Values

Channel	Color	Minimum	Maximum	K
Chemi	Gray Scale (Black on White)	0.00000554	0.00350	0
700	Gray Scale (Black on White)	0.0000458	0.120	0

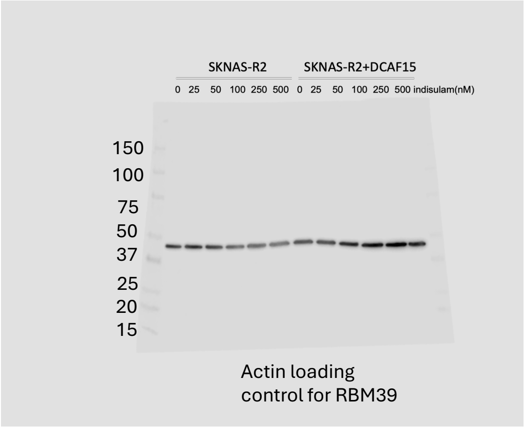

Image ID: 0001497_02
Acquire Time: Apr 26, 2021, 2:09:41 AM

Page 1

Images Table

#	Image ID	Acquire Time	Channels	Integration Times	Analysis	Image Name	Comment
1	0001497_02	Apr 26, 2021, 2:09:41 AM	700, Chemi	04:30 00:30	Manual	0001497_02	DCAF15 R2

Image Display Values

Channel	Color	Minimum	Maximum	K
Chemi	Gray Scale (Black on White)	0.0000000596	0.000168	0
700	Gray Scale (Black on White)	0.0000374	0.0195	0

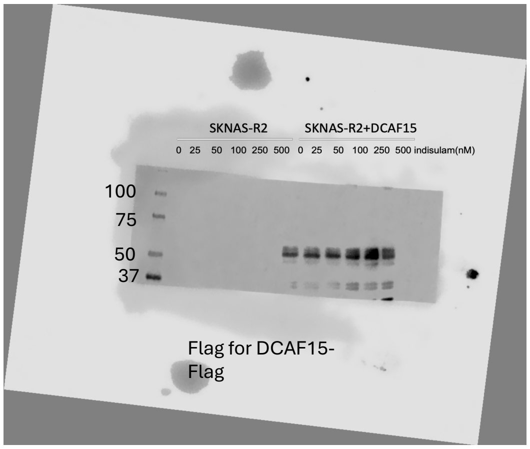

Image ID: 0001507_02
Acquire Time: Apr 30, 2021, 2:55:41 PM

Page 1

Images Table

#	Image ID	Acquire Time	Channels	Integration Times	Analysis	Image Name	Comment
1	0001507_02	Apr 30, 2021, 2:55:41 PM	700, Chemi	02:00 00:30	Manual	0001507_02	Actin for DCAF15 sknas-R2

Image Display Values

Channel	Color	Minimum	Maximum	K
Chemi	Gray Scale (Black on White)	0.000000775	0.00382	0
700	Gray Scale (Black on White)	0.0000373	0.0312	0

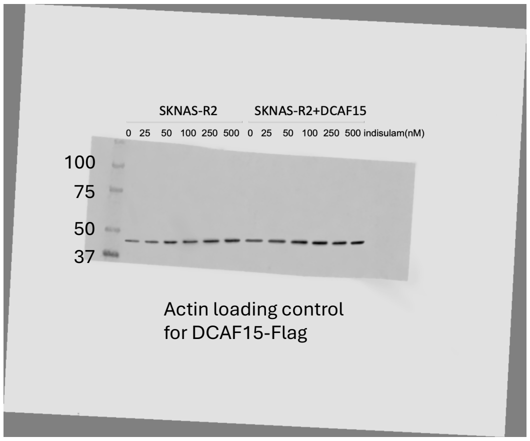

Uncropped blots for Supplementary Figure 5e

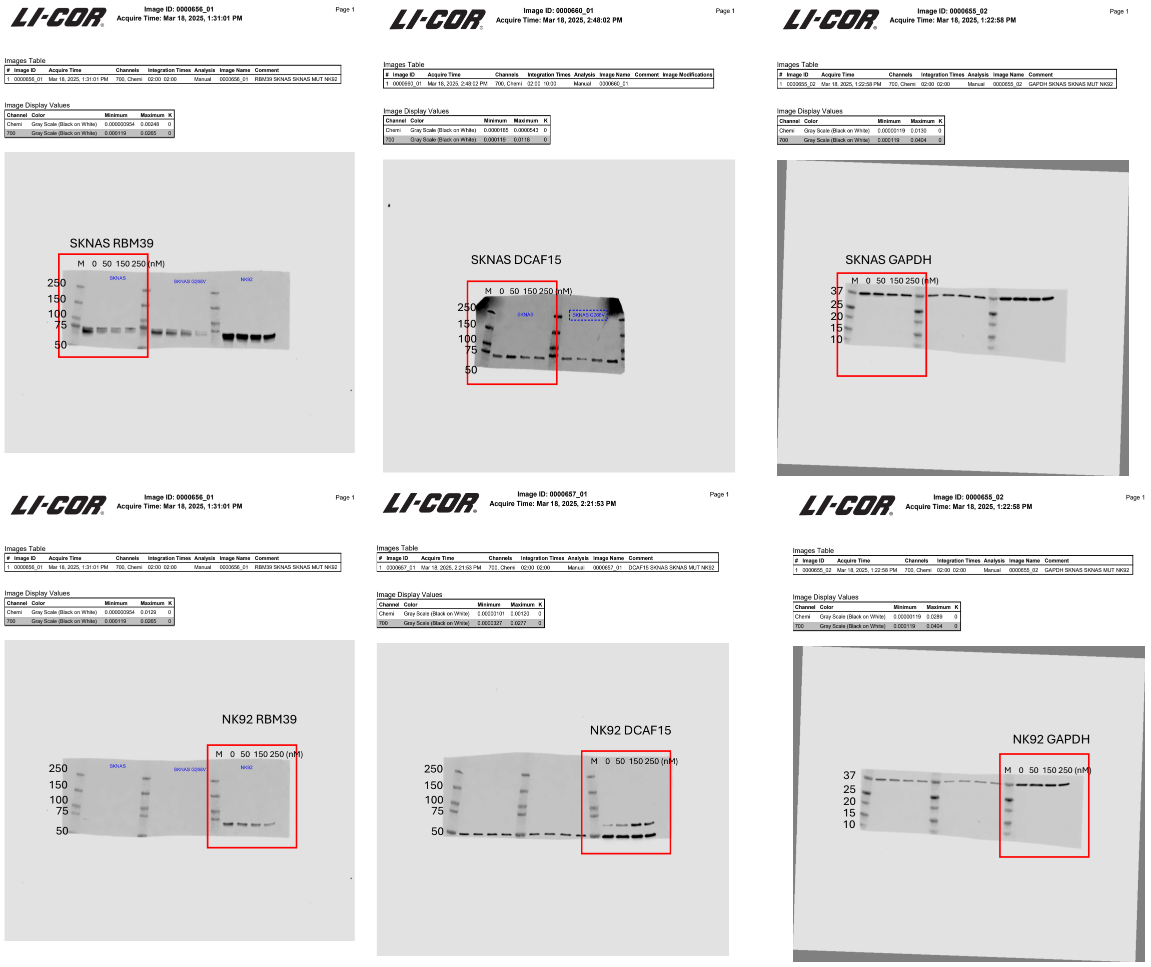

Supplement: Supplementary file 1 — Supplementary Information [file 41467_2025_63979_MOESM1_ESM.pdf]
