# Supplementary material for: RBM39 degrader invigorates innate immunity to eradicate neuroblastoma despite cancer cell plasticity
Source: Nat Commun. 2025 Sep 17;16:8287. doi: 10.1038/s41467-025-63979-x (PMC12443969; doi:10.1038/s41467-025-63979-x)
Supplement: Supplementary file 2 — Description of Additional Supplementary Files [file 41467_2025_63979_MOESM2_ESM.pdf]

Description of Additional Supplementary Files

Supplementary Data 1
Human transcription factor guide RNAs

Supplementary Data 2
Human epigenetics guide RNAs

Supplementary Data 3
Human kinome guide RNAs

Supplementary Data 4
Transcription factor CRISPR library screening results

Supplementary Data 5
Epigenetic CRISPR library screening results

Supplementary Data 6
Kinome CRISPR library screening results

Supplementary Data 7
RNA expression of kinases essential to parental SKNAS and the indisulam-resistant SKNAS R2 line

Supplementary Data 8
Cell clusters in Th-MYCN/ALKF1178L model treated with vehicle and indisulam
