# Supplementary material for: RBM39 degrader invigorates innate immunity to eradicate neuroblastoma despite cancer cell plasticity
Source: Nat Commun. 2025 Sep 17;16:8287. doi: 10.1038/s41467-025-63979-x (PMC12443969; doi:10.1038/s41467-025-63979-x)
Supplement: Supplementary file 11 — Reporting Summary [file 41467_2025_63979_MOESM11_ESM.pdf]

Corresponding author(s): Jun Yang
Last updated by author(s): Jul 15, 2025

Reporting Summary

Nature Portfolio wishes to improve the reproducibility of the work that we publish. This form provides structure for consistency and transparency in reporting. For further information on Nature Portfolio policies, see our Editorial Policies and the Editorial Policy Checklist.

Statistics

For all statistical analyses, confirm that the following items are present in the figure legend, table legend, main text, or Methods section.

n/a	Confirmed	
☐	☒	The exact sample size (*n*) for each experimental group/condition, given as a discrete number and unit of measurement
☐	☒	A statement on whether measurements were taken from distinct samples or whether the same sample was measured repeatedly
☐	☒	The statistical test(s) used AND whether they are one- or two-sided<br>*Only common tests should be described solely by name; describe more complex techniques in the Methods section.*
☒	☐	A description of all covariates tested
☒	☐	A description of any assumptions or corrections, such as tests of normality and adjustment for multiple comparisons
☐	☒	A full description of the statistical parameters including central tendency (e.g. means) or other basic estimates (e.g. regression coefficient) AND variation (e.g. standard deviation) or associated estimates of uncertainty (e.g. confidence intervals)
☐	☒	For null hypothesis testing, the test statistic (e.g. *F*, *t*, *r*) with confidence intervals, effect sizes, degrees of freedom and *P* value noted<br>*Give P values as exact values whenever suitable.*
☒	☐	For Bayesian analysis, information on the choice of priors and Markov chain Monte Carlo settings
☒	☐	For hierarchical and complex designs, identification of the appropriate level for tests and full reporting of outcomes
☐	☒	Estimates of effect sizes (e.g. Cohen's *d*, Pearson's *r*), indicating how they were calculated

Our web collection on statistics for biologists contains articles on many of the points above.

Software and code

Policy information about availability of computer code

Data collection	Bulk RNA-seq, ATAC-seq and CUT&TAG data were collected by using the Illumina HiSeq4000.<br>For scMultiome analysis, data were collected and processed using Cell Ranger ARC (v2.0, 10X Genomics)
Data analysis	For bulk RNA-seq analysis, Trim-Galore version 0.60, STAR v2.7, RSEM v1.31, R limma package version 3.42.2 and gsea2 version 2.2.3 and rMATS v4.1.0 were used. For ATAC-seq and CUT&TAG, BWA, Picard version 1.65, samtools version 1.3.1,MACS2, BEDtools, HOMER, IGV 2.3.82 were used. For scMultiome analysis, data were processed using Cell Ranger (10x Genomics). Seurat v5.0.0 was used for scRNA-seq analysis, with batch correction performed using Harmony v1.2. Visualization was conducted using ggplot2 v3.5.0 and dittoSeq v1.10. scATAC-seq were processed using Seurat v5.0.0 and Signac v1.12.0

For manuscripts utilizing custom algorithms or software that are central to the research but not yet described in published literature, software must be made available to editors and reviewers. We strongly encourage code deposition in a community repository (e.g. GitHub). See the Nature Portfolio guidelines for submitting code & software for further information.

Data

Policy information about availability of data

All manuscripts must include a data availability statement. This statement should provide the following information, where applicable:

- Accession codes, unique identifiers, or web links for publicly available datasets
- A description of any restrictions on data availability
- For clinical datasets or third party data, please ensure that the statement adheres to our policy

The RNA-seq data, ATAC-seq data, CUT&Tag data have been deposited in GEO database, which are publicly available now.
The GSE251920 SuperSeries is composed of the following SubSeries: link: https://www.ncbi.nlm.nih.gov/geo/query/acc.cgi?&acc=GSE251920
GSE251915 [CUT&Tag of H3K27Ac for SKNAS and SJNB14 models], https://www.ncbi.nlm.nih.gov/gds/?term=GSE251915
GSE251918 [RNA-seq for TH-MYCN/ALKF1178L and SJNB14 models], https://www.ncbi.nlm.nih.gov/geo/query/acc.cgi?acc=GSE251918
GSE164505 [RNA-seq for SIMA and SKNAS models, ATAC-seq for SKNAS model] , https://www.ncbi.nlm.nih.gov/gds/?term=GSE164505
GSE293865 [MultiOmics for Th-MYCN/ALKF1178L], Link: https://www.ncbi.nlm.nih.gov/geo/query/acc.cgi?acc=GSE293865
Target NBL data can be freely accessed through https://portal.gdc.cancer.gov/projects/TARGET-NBL. St Jude PCGP data can be freely accessed through https://stjude.cloud/. RNA-seq for SEQC neuroblastoma cohort (GSE62564) can be feely accessed through https://hgserver1.amc.nl/cgi-bin/r2/main.cgi."

Research involving human participants, their data, or biological material

Policy information about studies with human participants or human data. See also policy information about sex, gender (identity/presentation), and sexual orientation and race, ethnicity and racism.

Reporting on sex and gender	*Use the terms sex (biological attribute) and gender (shaped by social and cultural circumstances) carefully in order to avoid confusing both terms. Indicate if findings apply to only one sex or gender; describe whether sex and gender were considered in study design; whether sex and/or gender was determined based on self-reporting or assigned and methods used. Provide in the source data disaggregated sex and gender data, where this information has been collected, and if consent has been obtained for sharing of individual-level data; provide overall numbers in this Reporting Summary. Please state if this information has not been collected. Report sex- and gender-based analyses where performed, justify reasons for lack of sex- and gender-based analysis.*
Reporting on race, ethnicity, or other socially relevant groupings	*Please specify the socially constructed or socially relevant categorization variable(s) used in your manuscript and explain why they were used. Please note that such variables should not be used as proxies for other socially constructed/relevant variables (for example, race or ethnicity should not be used as a proxy for socioeconomic status). Provide clear definitions of the relevant terms used, how they were provided (by the participants/respondents, the researchers, or third parties), and the method(s) used to classify people into the different categories (e.g. self-report, census or administrative data, social media data, etc.) Please provide details about how you controlled for confounding variables in your analyses.*
Population characteristics	*Describe the covariate-relevant population characteristics of the human research participants (e.g. age, genotypic information, past and current diagnosis and treatment categories). If you filled out the behavioural & social sciences study design questions and have nothing to add here, write "See above."*
Recruitment	*Describe how participants were recruited. Outline any potential self-selection bias or other biases that may be present and how these are likely to impact results.*
Ethics oversight	*Identify the organization(s) that approved the study protocol.*

Note that full information on the approval of the study protocol must also be provided in the manuscript.

Field-specific reporting

Please select the one below that is the best fit for your research. If you are not sure, read the appropriate sections before making your selection.

☒ Life sciences ☐ Behavioural & social sciences ☐ Ecological, evolutionary & environmental sciences

For a reference copy of the document with all sections, see nature.com/documents/nr-reporting-summary-flat.pdf

Life sciences study design

All studies must disclose on these points even when the disclosure is negative.

Sample size	Experiments were designed to have enough sample sizes to obtain reliable results. We estimated the sample size considering no significant variation within each group of data. The principle of using the smallest sample size possible was adopted in planning the animal experiments. We estimated the sample size in order to detect a difference in averages of 2 standard deviations at the 0.05 level of significance with an 80% power. Figure 1a, mouse n=5. Figure 2a, mouse n=5 for control, mouse n=5 for treatment. Figure 5d, control mouse n=5, indisulam therapy mouse n=5, Gartisertib therapy mouse n=5, combination therapy mouse n=5. Figure 7b, NSG mouse n=5, Rag2-/- mouse n=6, C57BL6 mouse n=6. Figure 7d, vehicle mouse n=6.Figure 7e, indisulam mouse =6. Figure 7f, control mouse n=3, indisulam mouse n=3. Figure 8c, control mouse =3, indisulam mouse n=3. Figure 8 and 8e,, control mouse n=11, indisulam mouse n= 8, GD2 mouse n=6, combination mouse n=8.

	Figure 8f and 8g, vehivle =5, anti-GD2=5, indisulam =5, combination =5. Figure 8h and 8i, vehicle =6, TMZ=4, indisulam =6, anti-GD2 =6, combination =6. For in vitro experiments, unless indicated, at least three independent replicates and a minimum of two biological replicates were used for each experiment to ensure the reproducibility and to perform statistical analysis.
Data exclusions	No data exclusion
Replication	Data was obtained from three technical replicates and in at least two biological replicates. Independent biological replicates are shown in all figures.
Randomization	For in vitro experiments, all cell lines or biological samples were analyzed or treated in the same manner. For in vivo therapy, mice were enrolled in random into each treatment group.
Blinding	Experiments were not blinded in order to allow the investigators to have correct identification of samples and to ensure the correct data collection. In other hand, blinding strategy was applied to computational biologists who performed bioinformatic analyses and animal center staff who monitored tumor growth by ultrasound imaging.

Reporting for specific materials, systems and methods

We require information from authors about some types of materials, experimental systems and methods used in many studies. Here, indicate whether each material, system or method listed is relevant to your study. If you are not sure if a list item applies to your research, read the appropriate section before selecting a response.

Materials & experimental systems

n/a	Involved in the study	
☐	☒	Antibodies
☐	☒	Eukaryotic cell lines
☒	☐	Palaeontology and archaeology
☐	☒	Animals and other organisms
☒	☐	Clinical data
☒	☐	Dual use research of concern
☒	☐	Plants

Methods

n/a	Involved in the study	
☐	☒	ChIP-seq
☐	☒	Flow cytometry
☒	☐	MRI-based neuroimaging

Antibodies

Antibodies used

Anti-DCAF15 (St John's Laboratory, STJ194383, dilution 1:500). The therapeutic Hu 14.18 K322A anti-GD2 monoclonal antibody made in house. anti-RBM39 primary antibody (Sigma, cat#:HPA001591, RRID: AB_1079749;1:1000, anti-HSP90 Primary antibody (Santa Cruz: Cat#:SC13119, 1;1000 dilution, RRID#AB_675659), anti-GD2 (Sigma Aldrich. Catalog # MAB2052) or Isotype control mAb (Thermo Fisher Scientific, Catalog # 14-4724-85), anti-H3K27ac (Abcam, ab4729; RRID:AB_2118291),anti-CD3 PE/Dazzle594 (clone 17A2, BioLegend, lot# B307674, dilution 1:200), anti-CD45 PE/Cy5 (clone 30-F11, BioLegend, lot# B340539, dilution 1:200), anti-CD49b BV605 (clone HM[illegible]2S, BD Biosciences, lot# 1305998, dilution 1:200).

Validation

All antibodies except the therapeutic anti-GD2 mAb were purchased from commercial vendors and were validated by manufactures, other studies and/or in this study.
anti-DCAF15: https://stjohnslabs.com/anti-dcaf15-antibody-251-301-aa-stj194383/?srsltid=AfmBOorEa_1BGM78RbzBUs5uiLXyeu4GM_g4-1U5BMVTYJ_mKd6wbylx
anti-RBM39: https://www.sigmaaldrich.com/CA/en/product/sigma/hpa001591?srsltid=AfmBOooNfseYcNYb6obIUYON83HOAoll0h8LD-nTrarT-00scMcNPaSc
anti-HSP90: https://www.scbt.com/p/hsp-90alpha-beta-antibody-f-8?utm_id=22653289422&utm_source=google&utm_medium=cpc&utm_term=sc13119&gad_source=1&gad_campaignid=22653289422&gbraid=0AAAAAD8VVLeo_QufaSZe56KV1vASPtayo&gclid=EAIaIQobChMI9YPgtPOCjgMVqGtHAR2LtjoWEAAYAiAAEgJYtPD_BwE
anti-GD2: https://www.sigmaaldrich.com/CA/en/product/mm/mab2052?srsltid=AfmBOoozgbveKyLzPb1IYas1vTiIM_WNZd3GyTAmpYPJLIOIqXarUV2v
anti-H3K27Ac: https://www.abcam.com/en-us/products/primary-antibodies/histone-h3-acetyl-k27-antibody-chip-grade-ab4729?srsltid=AfmBOor1VRJwnd0M1zUL-kdwaMQPfAS4t0wZu8qkX-LjsLveLNKbxBRG
anti-CD3 PE/Dalle594: https://www.biolegend.com/fr-lu/products/pe-dazzle-594-anti-mouse-cd3-antibody-11946
anti-CD45 PE/Cy5: https://www.biolegend.com/en-ie/products/pe-cyanine5-anti-mouse-cd45-antibody-101
anti-CD49b BV605:https://www.bdbiosciences.com/en-us/products/reagents/flow-cytometry-reagents/research-reagents/single-color-antibodies-ruo/bv605-hamster-anti-mouse-cd49b-integrin-2.569508?tab=product_details

Eukaryotic cell lines

Policy information about cell lines and Sex and Gender in Research

Cell line source(s)

SIMA (DSMZ, ACC164)
SK-N-AS (ATCC, CRL-2137)
SK-N-AS-naive (This manuscript)

	SK-N-AS-indisulam R1 (This manuscript)<br>SK-N-AS-indisulam R2 (This manuscript)<br>SK-N-AS-indisulam R3 (This manuscript)
Authentication	Cell lines were authenticated by short tandem repeat (STR) using Promega PowerPlex 16 HS System once per month.
Mycoplasma contamination	PCR-based method was used for detection of Mycoplasma with LookOut Mycoplasma PCR Detection Kit (Sigma) and JumpStart Taq DNA Polymerase (Sigma) once per month to ensure cells were mycoplasma negative.
Commonly misidentified lines<br>(See ICLAC register)	No commonly misidentified cell lines were used in this study.

Animals and other research organisms

Policy information about studies involving animals; ARRIVE guidelines recommended for reporting animal research, and Sex and Gender in Research

Laboratory animals	CB17 SCID mice (CB17 scid, Taconic)<br>NOD.Cg-Prkdcscid Il2rgtm1Wjl/SzJ (NOD scid gamma (NSG, St Jude Children's Research Hospital, bred in house ARC)<br>C57BL/6J, The Jackson Laboratory, Strain #:000664, RRID:IMSR_JAX:000664<br>Rag2-/- (C57BL/6J-Rag2em3Lutzy/J), The Jackson Laboratory, Strain #:033526, RRID:IMSR_JAX:033526<br>TH-MYCN/ALKF1178L (this study).<br>Dbh-iCre; CAG-MYC (this study)
Wild animals	No wild animals were used in this study
Reporting on sex	Both genders of mouse were applied when using syngeneic Dbh-iCre/CAG-MYC models implanted into C57BL/6J and Rag2-/- mice, and TH-MYCN/ALKF1178L models. One gender of CB17 SCID or NSG mice were applied in this study.
Field-collected samples	No Field-collected samples were used in this study
Ethics oversight	All experiments that involved the use of mice were performed in accordance with the guidelines outlined by the St Jude Children's Research Hospital Institutional Animal Care and Use Committee (IACUC).

Note that full information on the approval of the study protocol must also be provided in the manuscript.

Plants

Seed stocks	*Report on the source of all seed stocks or other plant material used. If applicable, state the seed stock centre and catalogue number. If plant specimens were collected from the field, describe the collection location, date and sampling procedures.*
Novel plant genotypes	*Describe the methods by which all novel plant genotypes were produced. This includes those generated by transgenic approaches, gene editing, chemical/radiation-based mutagenesis and hybridization. For transgenic lines, describe the transformation method, the number of independent lines analyzed and the generation upon which experiments were performed. For gene-edited lines, describe the editor used, the endogenous sequence targeted for editing, the targeting guide RNA sequence (if applicable) and how the editor was applied.*
Authentication	*Describe any authentication procedures for each seed stock used or novel genotype generated. Describe any experiments used to assess the effect of a mutation and, where applicable, how potential secondary effects (e.g. second site T-DNA insertions, mosiacism, off-target gene editing) were examined.*

ChIP-seq

Data deposition

☒ Confirm that both raw and final processed data have been deposited in a public database such as GEO.

☒ Confirm that you have deposited or provided access to graph files (e.g. BED files) for the called peaks.

Data access links<br>*May remain private before publication.*	The reviewer access link: https://www.ncbi.nlm.nih.gov/geo/query/acc.cgi?&acc=GSE251920&token=sxopsaqanvahlqn<br>https://0-www-ncbi-nlm-nih-gov.brum.beds.ac.uk/geo/query/acc.cgi?acc=GSE164505
Files in database submission	GSE251915 [CUT&Tag of H3K27Ac for SKNAS and SJNB14 models]<br>GSE251918 [RNA-seq for TH-MYCN/ALKF1178L and SJNB14 models]<br>GSE164505 [RNA-seq for SIMA and SKNAS models, ATAC-seq for SKNAS model]
Genome browser session<br>(e.g. UCSC)	*Provide a link to an anonymized genome browser session for "Initial submission" and "Revised version" documents only, to enable peer review. Write "no longer applicable" for "Final submission" documents.*

Methodology

Replicates	two replicates per condition
Sequencing depth	For Cut&Tag, total number of reads is from 38 to 78 million, uniquely mapped reads range from 6 to 12 million. The length of reads is 36bp. they are paired-end.
Antibodies	H3K27ac (Abcam, ab4729; RRID:AB_2118291)
Peak calling parameters	Only properly paired uniquely mapped reads were extracted by samtools (version 1.3.1 parameters used were -q 1 -f 2 -F 1804). MACS2 (version 2.1.1 20160309) was used to call narrow peaks using 'macs2 callpeak -t cut_tag_file -f BEDPE -q 0.05 --keep-dup all' .
Data quality	Data quality check criteria require at least 2 million uniquely mapped reads /library and 50000 narrow peaks with FDR < 5% per samples
Software	BWA, Picard version 1.65, samtools version 1.3.1,MACS2, BEDtools, HOMER, IGV 2.3.82 were used.

Flow Cytometry

Plots

Confirm that:

☒ The axis labels state the marker and fluorochrome used (e.g. CD4-FITC).

☒ The axis scales are clearly visible. Include numbers along axes only for bottom left plot of group (a 'group' is an analysis of identical markers).

☒ All plots are contour plots with outliers or pseudocolor plots.

☒ A numerical value for number of cells or percentage (with statistics) is provided.

Methodology

Sample preparation	Tumors were excised, mechanically dissociated and transferred to basal medium containing 0.1% Collagenase Type IV (Worthington Biochemical Corporation , Cat#CLS-4) and 150 mg/mL DNase I (Promega, Cat# M6101), incubated for 30-minute shaking at 37°C, then passed through a 70-µm nylon mesh. Cells were treated with ACK red blood cell lysis buffer and resuspended in PBS prior to further analysis.
Instrument	Data were acquired with a Cytek Aurora 4-laser spectral flow cytometer.
Software	Data were analyzed in FlowJo v10 (Treestar).
Cell population abundance	N/A. Cells were not sorted.
Gating strategy	For all samples, preliminary FSC/SSC, single cell gates, Live/Dead gates were applied before gating on the downstream populations of interest. Positive and negative populations were determined by single color controls and/or isotype controls.

☒ Tick this box to confirm that a figure exemplifying the gating strategy is provided in the Supplementary Information.
